# Validation of Candidate Phospholipid Biomarkers of Chronic Kidney Disease in Hyperglycemic Individuals and Their Organ-Specific Exploration in Leptin Receptor-Deficient db/db Mouse

**DOI:** 10.3390/metabo11020089

**Published:** 2021-02-03

**Authors:** Jialing Huang, Marcela Covic, Cornelia Huth, Martina Rommel, Jonathan Adam, Sven Zukunft, Cornelia Prehn, Li Wang, Jana Nano, Markus F. Scheerer, Susanne Neschen, Gabi Kastenmüller, Christian Gieger, Michael Laxy, Freimut Schliess, Jerzy Adamski, Karsten Suhre, Martin Hrabe de Angelis, Annette Peters, Rui Wang-Sattler

**Affiliations:** 1 Research Unit of Molecular Epidemiology, Helmholtz Zentrum München, 85764 Neuherberg, Germany; jialing.huang@helmholtz-muenchen.de (J.H.); marcela.covic@helmholtz-muenchen.de (M.C.); martina.troll@helmholtz-muenchen.de (M.R.); jonathan.adam@helmholtz-muenchen.de (J.A.); wlrst@126.com (L.W.); christian.gieger@helmholtz-muenchen.de (C.G.); 2 Institute of Epidemiology, Helmholtz Zentrum München, 85764 Neuherberg, Germany; cod.huth@gmail.com (C.H.); jana.nano@helmholtz-muenchen.de (J.N.); peters@helmholtz-muenchen.de (A.P.); 3 German Center for Diabetes Research (DZD), 85764 München-Neuherberg, Germany; hrabe@helmholtz-muenchen.de; 4 Research Unit of Molecular Endocrinology and Metabolism, Helmholtz Zentrum München, 85764 Neuherberg, Germany; zukunft@vrc.uni-frankfurt.de (S.Z.); adamski@helmholtz-muenchen.de (J.A.); 5 Centre for Molecular Medicine, Institute for Vascular Signaling, Goethe University, 60323 Frankfurt am Main, Germany; 6 Metabolomics and Proteomics Core Facility, Helmholtz Zentrum München, 85764 Neuherberg, Germany; prehn@helmholtz-muenchen.de; 7 Liaocheng People’s Hospital—Department of Scientific Research, Shandong University Postdoctoral Work Station, Liaocheng 252000, China; 8 Institute of Experimental Genetics, Helmholtz Zentrum München, 85764 Neuherberg, Germany; markus@scheerer-home.de (M.F.S.); susanne.neschen@mail.com (S.N.); 9 Bayer AG, Medical Affairs & Pharmacovigilance, 13353 Berlin, Germany; 10 Sanofi Aventis Deutschland GmbH, Industriepark Hoechst, 65929 Frankfurt am Main, Germany; 11 Institute of Computational Biology, Helmholtz Zentrum München, 85764 Neuherberg, Germany; g.kastenmueller@helmholtz-muenchen.de; 12 Institute of Health Economics and Health Care Management, Helmholtz Zentrum München, 85764 Neuherberg, Germany; michael.laxy@helmholtz-muenchen.de; 13 Profil, 41460 Neuss, Germany; Freimut.Schliess@profil.com; 14 Department of Biochemistry, Yong Loo Lin School of Medicine, National University of Singapore, Singapore 117597, Singapore; 15 Chair of Experimental Genetics, Center of Life and Food Sciences Weihenstephan, Technische Universität München, 85353 Freising, Germany; 16 Department of Physiology and Biophysics, Weill Cornell Medical College in Qatar (WCMC-Q), Education City, Qatar Foundation, Doha P.O. Box 24144, Qatar; karsten@suhre.fr

**Keywords:** chronic kidney disease, prediabetes and type 2 diabetes, diabetic nephropathy, reduced kidney function, leptin receptor-deficient mouse, high-fat-diet, liver, lungs, metabolomics

## Abstract

Biological exploration of early biomarkers for chronic kidney disease (CKD) in (pre)diabetic individuals is crucial for personalized management of diabetes. Here, we evaluated two candidate biomarkers of incident CKD (sphingomyelin (SM) C18:1 and phosphatidylcholine diacyl (PC aa) C38:0) concerning kidney function in hyperglycemic participants of the Cooperative Health Research in the Region of Augsburg (KORA) cohort, and in two biofluids and six organs of leptin receptor-deficient (db/db) mice and wild type controls. Higher serum concentrations of SM C18:1 and PC aa C38:0 in hyperglycemic individuals were found to be associated with lower estimated glomerular filtration rate (eGFR) and higher odds of CKD. In db/db mice, both metabolites had a significantly lower concentration in urine and adipose tissue, but higher in the lungs. Additionally, db/db mice had significantly higher SM C18:1 levels in plasma and liver, and PC aa C38:0 in adrenal glands. This cross-sectional human study confirms that SM C18:1 and PC aa C38:0 associate with kidney dysfunction in pre(diabetic) individuals, and the animal study suggests a potential implication of liver, lungs, adrenal glands, and visceral fat in their systemic regulation. Our results support further validation of the two phospholipids as early biomarkers of renal disease in patients with (pre)diabetes.

## 1. Introduction

Diabetic nephropathy is the leading cause of chronic kidney disease (CKD) and end-stage kidney disease [1]. Early screening of persons with prediabetes or type 2 diabetes (T2D) for CKD predisposition can increase the opportunity to effectively prevent and manage this microvascular complication of diabetes in the framework of more personalized diabetes management [2]. However, targeted screening is important to assure the efficient allocation of health care resources [3].

Traditional markers for CKD are unable to accurately predict the development of CKD in individuals with T2D. Urinary albumin-to-creatinine ratio (UACR) and estimated glomerular filtration rate (eGFR) were found to be the most important variables to predict the onset and progression of early CKD in individuals with T2D in a large randomized clinical trial with a follow-up period of 5.5 years. However, even when combined with age and sex (i.e., a set of four clinical variables: age, sex, eGFR, and UACR), their predictive ability was found to be modest with an externally validated c-statistic of 0.68 [4].

Metabolomics is still a relatively new approach for studying metabolic changes connected to disease development and progression, as well as for finding predictive biomarkers to enable early interventions [5,6,7,8]. Using baseline metabolite profiles of a population-based Cooperative Health Research in the Region of Augsburg (KORA) cohort, we have recently discovered two candidate metabolite biomarkers (sphingomyelin (SM) C18:1 and phosphatidylcholine diacyl (PC aa) C38:0) for incident CKD that were specific for hyperglycemic individuals with prediabetes or T2D [9]. SM C18:1 and PC aa C38:0 were identified from 125 targeted metabolites through three-step feature selection that included multivariate logistic regression adjustment, priority-lasso filtering and stepwise Akaike information criterion selection. These two metabolites were in combination with five clinical variables (age, total cholesterol, fasting glucose, eGFR, and UACR) identified as the best set of predictors for incident CKD. Their predictive performance yielded a mean area value under the receiver operating characteristic curve of 0.857 and outperformed the performance of 14 known risk factors of CKD [9]. However, physiological mechanisms leading to circulatory accumulation of these new candidate biomarkers during the pathogenesis of diabetes-related CKD have not yet been delineated.

Altered serum levels of phospholipids in hyperglycemic individuals under higher risk of developing CKD [9] might indicate early alterations not only in the kidneys [10] but also other organ systems [11]. Insufficient elimination of a large number of potentially toxic organic metabolites from the vascular bed into the urine during CKD affects multiple body systems and organs [12]. Biological exploration of the emerging biomarkers is necessary towards a better understanding of the complex metabolic interactions between the circulatory, musculoskeletal and respiratory systems in CKD and their potential clinical application in diagnostics [12]. Moreover, animal models reflecting the pathogenetic evolution of diabetes-related CKD allow for direct analysis of organ-specific metabolite patterns during aggravation of the disease. The leptin-receptor deficient mouse model (db/db) was shown to exhibit a very consistent and robust increase in albuminuria and mesangial matrix expansion. It is therefore a well-established model for human diabetic nephropathy [13,14].

In this study, we evaluated the associations of SM C18:1 and PC aa C38:0 with eGFR values and risk of CKD with the recently generated targeted metabolites profiles of KORA FF4 study in participants with hyperglycemia. Furthermore, we examined creatinine, SM C18:1, and PC aa C38:0 levels in two biofluids (plasma, urine) and six tissues (liver, lungs, adrenal glands, adipose tissue, cerebellum, and testis) of db/db and wild type (WT) mice under high-fat diet (HFD) to explore organ-specific variations and discuss the potential link to various clinical symptoms. Our findings provide first insights into the potential involvement of several organs in the systemic accumulation of these metabolite biomarkers during CKD pathogenesis.

## 2. Results

### 2.1. Associations of the Two Metabolites with eGFR and CKD in Hyperglycemic Individuals

#### 2.1.1. Characteristics of the KORA FF4 Study Participants

Among 1907 eligible KORA FF4 participants, 168 individuals had CKD (8.8%). As expected, hyperglycemic participants were diagnosed more frequently to have CKD (16.3%) than individuals with normal glucose tolerance (NGT) (6.1%) (Table 1). The cases of CKD in hyperglycemic and NGT groups were significantly older and had significantly higher values of creatinine and UACR than non-CKD individuals in each group. The self-reported intake of antihypertensive and lipid-lowering medication was also significantly higher in cases of CKD. Compared to non-CKD individuals, the cases of CKD in the NGT group had also significantly higher values of BMI, triglycerides, glycated hemoglobin (HbA_1C_), fasting glucose, and 2-h post-load glucose (2-h glucose) (Table 1).

#### 2.1.2. Inverse Associations of the Two Metabolites with eGFR in Hyperglycemic Individuals

The inverse association between eGFR and the concentrations of SM C18:1 and PC aa C38:0 in hyperglycemic individuals was significant in all three weighted regression models (adjusted for imbalanced, basic and full model covariates) after applying inverse probability weighting (IPW). For example, a SD increase in the ln-transformed SM C18:1 concentration was associated with a 1.76 mL/min/1.73 m^2^ decrease in eGFR in the full model (*p* = 2.499 × 10^−3^; Table 2).

#### 2.1.3. Associations of the Two Metabolites with CKD Are Specific for Hyperglycemia

**Figure 1:**
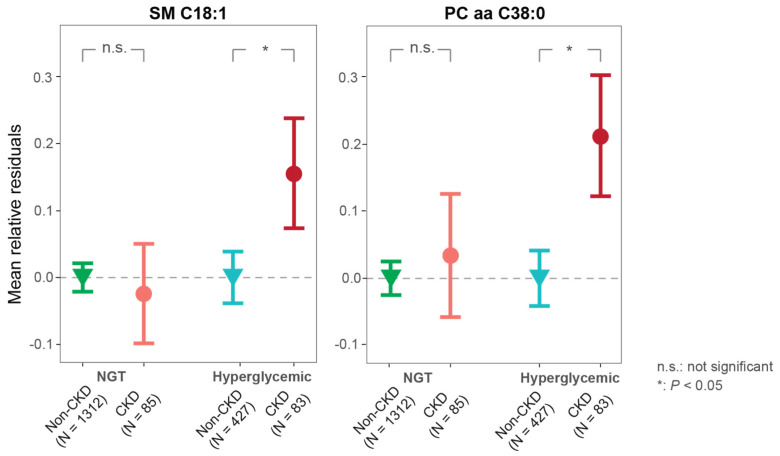
Stratified associations of the two candidate metabolites with CKD according to hyperglycemic and normoglycemic status. Mean relative residuals (with standard errors) of SM C18:1 and PC aa C38:0 for non-CKD and CKD in hyperglycemic and NGT individuals are shown, respectively. Metabolite relative residuals were calculated with linear regression models adjusted for age, sex, BMI, systolic blood pressure, triglyceride, total cholesterol, HDL cholesterol, HbA_1C_, smoking status, the use of lipid-lowering, antihypertensive medication, and urinary albumin-to-creatinine ratio. *p* values were calculated with multivariable logistic regression using CKD as outcome and adjusting covariates mentioned above. Abbreviations: CKD, chronic kidney disease; SM, sphingomyelin; PC aa, phosphatidylcholine diacyl; NGT, normal glucose tolerance.

The CKD cases with hyperglycemia had higher relative concentrations of the two metabolites (SM C18:1, PC aa C38:0) than non-CKD individuals (Figure 1). The concentrations of SM C18:1 and PC aa C38:0 were significantly positively associated with CKD in hyperglycemic individuals in all three models after IPW (Table 3). One SD increase in the ln-transformed SM C18:1 or PC aa C38:0 concentration was associated with a 99% or 71%, respectively, increased odds of CKD in hyperglycemic participants (full model *p* = 4.482 × 10^−4^ and 1.578 × 10^−3^, respectively, Table 3).

As a sensitivity analysis, we tested the associations of the two metabolites with CKD in normoglycemic KORA participants. Both SM C18:1 and PC aa C38:0 were not significantly associated with CKD in NGT individuals in all three models after IPW (Table 3). As shown in Figure 1, normoglycemic individuals with diagnosed CKD did not show any significant differences in their relative metabolite concentration when compared to healthy NGTs. These results further confirmed that the risk associations of the two lipids are specific for hyperglycemia.

### 2.2. Organ-Specific Trends of the Candidate Biomarkers in Diabetic Mice

#### 2.2.1. Characteristics of the Mouse Model

Organ trends of the two phospholipids were explored in the db/db mouse model that mimics the early human CKD development. After 5 weeks of HFD, the 8-week-old db/db mice were obese and had significantly higher heart, kidney and liver weight when compared with WT controls of the same age and diet (Table 4). Furthermore, their blood levels of glucose, insulin, cholesterol, and C-reactive protein were significantly higher confirming that db/db mice developed hyperglycemia, dyslipidemia and inflammation.

Their significantly elevated kidney weight indicated renal hypertrophy, which occurs in the early stage of diabetic nephropathy development [15] and is one of the early markers of morphological changes in renal tissue [16]. It has been shown that 8-week old diabetic mice present glomerular hypertrophy and significantly bigger glomerular tuft surface area compared to nondiabetic mice [17]. Glomerular hyperfiltration and hypertrophy are early features of diabetic nephropathy [15].

#### 2.2.2. Analysis of Creatinine in Eight Murine Tissues

Creatinine concentration in biofluids (plasma, urine) and organs (liver, lungs, adrenal gland, visceral adipose tissue, testis, cerebellum) was determined by targeted metabolomics. In plasma, creatinine was also measured with clinical chemistry. Pearson’s correlation coefficient of plasma creatinine concentrations measured with both methods was 0.923 (*p*-value = 6.938 × 10^−9^), showing a very high correlation between clinical chemistry- and mass spectrometry (MS)-based methods.

In addition to plasma, significantly lower values of creatinine were also detected in the urine, liver and lungs of db/db mice (Table 5). Our observation of approximately 40% lower plasma creatinine (Table 4) and its negative trend in the urine of db/db mice suggests impaired creatine biosynthesis, protein catabolism and glomerular hyperfiltration.

Taken together, our 8-week old db/db mice fed with HFD during 5 weeks reflected characteristic changes of early diabetic nephropathy, such as glomerular hyperfiltration and hypertrophy, as evidenced by significantly lower plasma and urinary creatinine levels and higher kidney weight. Moreover, their phenotypic and metabolic data show obesity, hyperglycemia, dyslipidemia, and inflammation, confirming previous reports about insulin resistance and fatty liver (steatosis) in db/db mice of similar age [13,14,18,19,20].

#### 2.2.3. Organ-Specific Trends of the Two Metabolites

**Figure 2:**
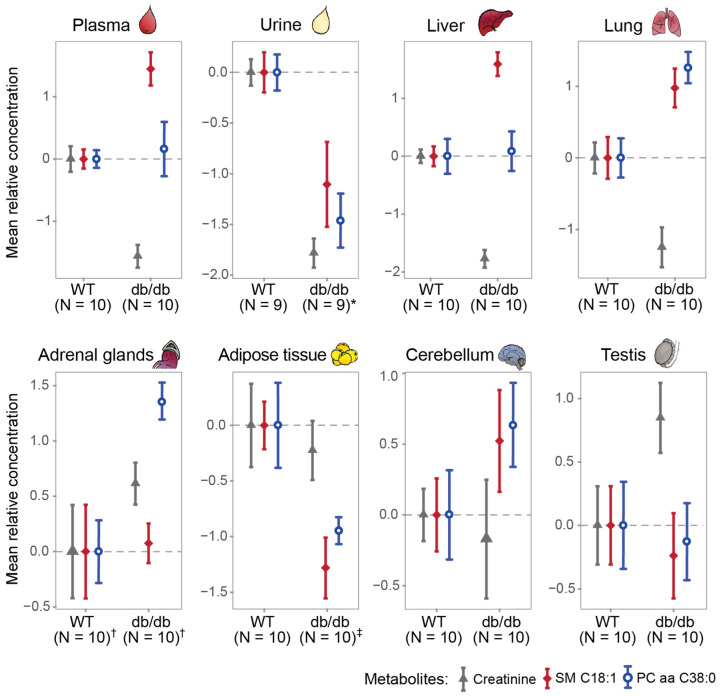
Analysis of creatinine and two candidate metabolites in murine biofluids and tissues. Mean relative concentrations (with standard errors) of the metabolites (creatinine, SM C18:1, and PC aa C38:0) in murine plasma, urine, liver, lung, adrenal tissue, adipose tissue, cerebellum, and testis. * N = 7 in db/db for SM C18:1. ^†^ For creatinine, N = 9 in db/db, N = 9 in WT. ^‡^ N = 9 in db/db for creatinine. Abbreviations: db/db, leptin receptor-deficient mouse model; WT, wild type mice; SM, sphingomyelin; PC aa, phosphatidylcholine diacyl.

As compared to WT mice, significantly higher concentrations of both SM C18:1 and PC aa C38:0 were found in the lungs of db/db mice, whereas significantly lower concentrations were found in urine and adipose tissue (Figure 2, Table 5). Furthermore, SM C18:1 was significantly accumulated in plasma (*p* = 3.160 × 10^−4^) and liver (*p* = 1.288 × 10^−5^), whereas PC aa C38:0 was significantly higher in adrenal glands (*p* = 9.695 × 10^−4^, Table 5) of db/db mice. The concentrations of both metabolites in cerebellum and testis were comparable (Table 5).

## 3. Discussion

According to the natural history of diabetic nephropathy, the early stage displays normal kidney function (normal GFR) and is clinically unsuspicious. It is followed by a transient period of glomerular hyperfiltration (increased GFR) that later normalizes and slowly decreases towards a steep GFR decline at a relatively later stage [21]. Our initial discovery in the longitudinal human cohort showed predictive effects of elevated serum levels of SM C18:1 and PC aa C38:0 for incident CKD in hyperglycemic individuals with normal baseline kidney function [9]. The finding of this animal and cross-sectional human study is that these metabolites associate with further stages of hyperglycemia-related CKD evolution including (i) early changes characterized with glomerular hyperfiltration (8-week-old db/db mice) and (ii) later changes characterized with reduced eGFR (KORA FF4 study).

This cross-sectional KORA FF4 study revealed significant associations between serum levels of SM C18:1 and PC aa C38:0 with decreased eGFR in individuals with prediabetes or T2D. Their associations with kidney function were independent of systolic blood pressure, blood lipids, HbA_1C_, and UACR suggesting that these two candidate phospholipids biomarkers are independent risk factors for CKD. Both metabolites, SM C18:1 and PC aa C38:0, are phospholipids that are known to regulate inflammation and fibrosis and their alterations in diabetes and metabolic syndrome occur in multiple body systems [11]. Besides hyperglycemia-related CKD [9], metabolomics studies have revealed that plasma PC aa C38:0 was positively associated with coronary artery disease mortality [22] and systemic alterations in SM levels were also predictive of T1D [23], T2D [24], and myocardial infarction [25]. As these outcomes are risk factors or subsequent outcomes for hyperglycemia-related CKD, further studies are necessary to provide insights into the disease-specificity of emerging phospholipid biomarkers before their application in clinical diagnostics. Since not all patients with diabetes develop CKD and not all patients with CKD follow the same disease trajectory, it is also important to explore their mechanisms of actions for better patient stratification and to accelerate targeted screening programs.

Glomerular hyperfiltration is a hallmark of kidney dysfunction in diabetes. The flow-related effects of glomerular and tubular changes caused by glomerular hyperfiltration-related mechanical stress play a major role in the pathogenesis of the glomerular disease, and reduction of hyperfiltration is a crucial therapeutic target in diabetes-induced CKD [26]. In young diabetic mice (6–10 weeks), exert supraphysiological GFR and increased creatinine clearance have been reported [17,27]. As a potential effect of glomerular hyperfiltration in our 8-week-old db/db mice, we observed lower plasma and urinary levels of creatinine. Creatinine is a toxic byproduct of phosphocreatine metabolism and is excreted by glomerular filtration and proximal tubular secretion with little to no reabsorption. Besides the plasma and urine in our db/db mice, lower concentrations of creatinine were also found in the liver and lungs, which could be explained by reduced creatine biosynthesis and/or phosphocreatine energy metabolism in skeletal muscle and other organs. The influence of known factors affecting serum creatinine values (age, sex, ethnicity, muscle mass, protein diet, and intake of drugs [28]) was minimal as these factors were controlled for in our mouse study. Diabetic mice display skeletal mass reduction already at 5 weeks of age and before T2D onset [29] and low serum creatinine in T2D patients indicates muscle loss and predicts T2D independently of glomerular filtration [30,31]. Taken together, creatinine measurements in our 8-week-old db/db mice are suggestive of not only altered kidney function, e.g., glomerular hyperfiltration, but also high-energy phosphate metabolism.

Our db/db mice displayed significantly higher levels of both metabolites, SM C18:1 and PC aa C38:0, in the lungs than WT mice. This could indicate lung dysfunction as PCs and SMs are key components of pulmonary surfactant and their dysregulation was linked with respiratory failure [32]. The db/db mice are prone to pulmonary edema [33] and asthma-related symptoms such as airway hyperresponsiveness [34]. Sphingomyelin synthase 2 (SMS2) deficiency attenuates inflammation and ameliorates recovery after lung injury in mice [35]. Lung dysfunction is common, but clinically less managed, comorbidity in patients with CKD [36]. Despite some earlier and controversial evidence on better adult respiratory distress syndrome (ARDS) survival in T2D patients, it has been urged to investigate lung dysfunction in T2D patients [37].

**Figure 3:**
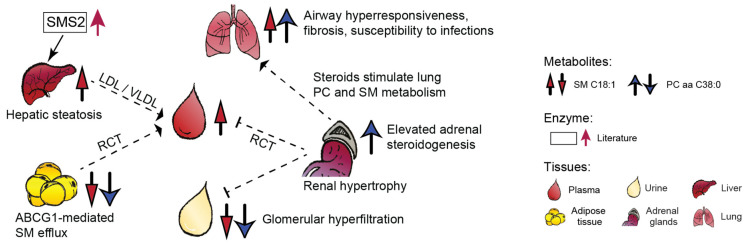
Organ-specific trends of SM C18:1 and PC aa C38:0 in a mouse model of diabetic nephropathy and potential interorgan crosstalk inferred from literature (interrupted lines, references in discussion). Abbreviations: ABCG1, ATP-binding cassette subfamily G member 1; RCT, reverse cholesterol transport; SMS2, sphingomyelin synthase 2; VLDL, very-low-density lipoprotein; LDL, low-density lipoprotein; SM, sphingomyelin; PC, phosphatidylcholine.

The epididymal adipose tissue in db/db mice displayed lower concentrations of SM C18:1 and PC aa C38:0 (Figure 3). In line with our findings, reduced adipose tissue levels of certain SMs and PCs have also been detected in 30-week old db/db mice [38]. The phospholipid metabolism in white adipose tissue and residing macrophages of obese animals is largely perturbed [39]. We speculate that the lower adipose levels of SM C18:1 and PC aa C38:0 could be due to increased efflux of SM- or PC-containing lipoproteins by the ATP-binding cassette transporter ABCG1 [40] that is upregulated in obese mice [41].

Higher hepatic levels of SM C18:1 in db/db mice could be the consequence of fatty liver related upregulation in SMS2 activity [42], which determines hepatic and plasma SM values [43]. SMS2 activity promotes fatty acid uptake and liver steatosis [42], whereas SMS2 deficiency prevents HFD-induced liver steatosis [44] and increases insulin sensitivity [45]. The liver is the central hub of phospholipid synthesis and recycling via lipoprotein particles such as LDL/VLDL (approx. 70% of plasma SMs) and HDL (30%) (Figure 3).

Our observation of higher concentration of PC aa C38:0 in the adrenal glands might be related with reduced biosynthesis of polyunsaturated fatty acids in adrenals of db/db mice [46]. These mice also display an increased synthesis of adrenal steroids [19], which can stimulate PC synthesis in the lungs [47] (Figure 3).

Biofluids such as blood and urine provide insights into interorgan metabolic crosstalk and kidney activity, respectively. Similarly to creatinine, the lower urinary levels of SM C18:1 and PC aa C38:0 in db/db mice may reflect altered glomerular filtration as well as phospholipid accumulation in the kidney tissue as was shown in HFD-fed db/db mice [48]. SMs accumulate in the glomeruli of diabetic and HFD-fed mice might promote CKD [49]. Diabetic kidney disease in db/db mice manifests around 8 weeks of age with albuminuria and increased glomerular surface area, resembling the early stage of human diabetic nephropathy, and is followed by a progressive increase in mesangial matrix and hypertrophy [13,50]. The kidneys modulate HDL metabolism and their early dysfunction could impair reverse cholesterol transport and additionally contribute to lower urinary concentrations of the two phospholipids (Figure 3). In summary, this detailed assessment of two biofluids and six tissues in a well-characterized mouse model of diabetic nephropathy indicates altered levels of SM C18:1 and PC aa C38:0 in the liver, lungs, adrenal gland, adipose tissue, and urine. Of these, the lungs appear especially interesting due to phospholipid implication in various pulmonary diseases and injuries [51]. At the current stage of knowledge, it is unclear but possible (based on literature) that these organs could also contribute to the circulatory regulation of SM C18:1 and PC aa C38:0.

This study has several limitations and advantages. Limited availability of the mouse data did not allow us to analyze kidney tissue nor validate metabolite profiles by histological analysis. Compared with humans, the difference in the genetic background of db/db mice that causes hyperglycemia and diabetic nephropathy may confound metabolite profiles. Therefore, multiorgan contribution to systemic dysregulation of SM C18:1 and PC aa C38:0 and their potential functional implication in kidney function (by feeding experiments in diabetic mouse models) require further investigations. One of strengths of our study is the validation of two candidate biomarkers of incident CKD not only in a cross-sectional human study, but also in multiorgan mouse models with hyperglycemia and obesity. Our study provides first insights into multistage CKD association, early stage characterized with glomerular hyperfiltration (8-week-old db/db mice), and later stage characterized with reduced eGFR (KORA FF4 study), as well as potential multiorgan contribution to circulatory regulation of the two phospholipid metabolites for CKD.

## 4. Materials and Methods

### 4.1. Study Participants, Outcome Definition

The KORA FF4 study was conducted in the area of Augsburg, Southern Germany. All study participants gave written informed consent. The KORA study was approved by the ethics committee of the Bavarian Medical Association, Munich, Germany.

Individuals with hyperglycemia and NGT were classified according to fasting glucose and 2-h glucose values using the World Health Organization diagnostic criteria. Hyperglycemic group comprised participants with prediabetes and newly diagnosed T2D (i.e., fasting glucose ≥ 110 mg/dL and/or 2-h-glucose ≥ 140 mg/dL), as well as known T2D that was diagnosed by physician validated self-reporting and/or current use of antidiabetes agents [8].

We examined 2218 individuals who had metabolite measurements and excluded 311 participants in the analysis including (1) nonfasting samples (*n* = 15); (2) missing eGFR, UACR, or covariate values (*n* = 37); (3) diagnosis for type 1 diabetes (*n* = 5), unclear type of diabetes mellitus (*n* = 69) or age equal to or greater than 85 (*n* = 23) or self-reported use of antidiabetic medication (*n* = 162). The remaining dataset comprised 510 hyperglycemic participants and 1397 individuals with NGT (Table 1). The hyperglycemic individuals were used to study the associations of eGFR and CKD with the two metabolites. The NGT individuals served as a sensitivity analysis of the associations of CKD with the two metabolites.

The eGFR was calculated from serum creatinine (mg/dl) (IDMS standardized values) using the Chronic Kidney Disease Epidemiology Collaboration (CKD-EPI) equation [52]. CKD was defined as an eGFR < 60 mL/min/1.73 m^2^ [53].

### 4.2. Mouse Study

**Figure 4:**
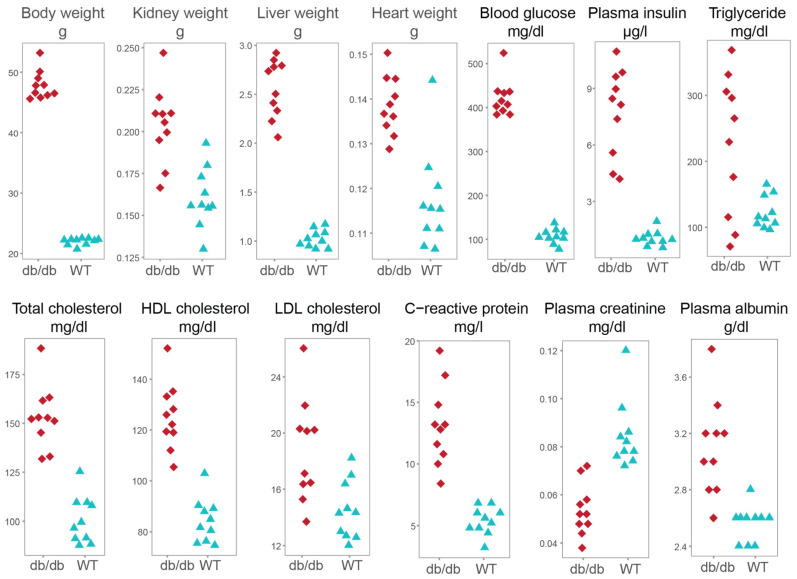
Scatter plots of phenotypic and metabolic variables in db/db and wild type mice fed with a high-fat diet. Abbreviations: HDL, high-density lipoprotein; LDL, low-density lipoprotein; db/db, leptin receptor-deficient mouse model; WT, wild type mice.

We used male 8-week (±3 d) old WT mice (*n* = 10) and db/db mice (BKS.Cg-*Dock7^m^*+/+ *Lepr^db^*/J, *n* = 10, Figure 4). The animals were bred and housed in a temperature- and humidity-controlled environment in compliance with FELASA (the Federation of Laboratory Animal Science Associations) protocols [54]. Animal experiments were approved by the District Government of Upper Bavaria (Regierung von Oberbayern, Gz.55.2-1-54-2531-70-07, 55.2-1-2532-153-11).

From an age of 3 weeks, all mice were fed with HFD (S0372-E010, ssniff Spezialdiäten, Soest, Germany) [54]. After receiving vehicle (5% solutol and 95% hydroxyethylcellulose), all mice were fasted for 4 h before biofluid and organ collection. Urine was collected individually with absorbing tissue pads. Blood samples were collected from lateral tail veins. Liver, epidydimal adipose tissue, cerebellum, lung, adrenal, and testis samples were immediately dissected and freeze-clamped after sacrification with an isoflurane overdose [54]. All samples were stored at −80 °C until further analyses.

### 4.3. Metabolite Quantification and Normalization

Serum samples from participants in the KORA FF4 study were measured with the Absolute*IDQ*^TM^ p180 Kit (BIOCRATES Life Sciences AG, Innsbruck, Austria). Metabolite concentrations were adjusted for plate normalization factors (NFs) to minimize the plate effect. For each metabolite, the plate NFs were calculated by dividing the mean of reference samples in each plate with the mean of all reference samples in all measured plates. Metabolite concentrations were natural-log transformed and scaled to a mean value of zero and standard deviation (SD) of one to ensure comparability between the metabolites.

In the mouse study, creatinine, SM C18:1 and PC aa C38:0 values in plasma, liver, lung, adrenal glands, adipose tissue, cerebellum, and testis samples were determined with the Absolute*IDQ*^TM^ p180 Kit (BIOCRATES Life Sciences AG, Innsbruck, Austria) and in urine with the Absolute*IDQ*^TM^ p150 Kit (BIOCRATES). Tissue homogenization, extraction solvents, assay preparation, and LC-MS/MS measurements have been described elsewhere [55]. Since each tissue sample from db/db and WT mice was measured on the same kit plate, we did not conduct plate correction. Metabolite concentrations were natural-log transformed and then scaled to a mean value of zero and SD of one for each tissue.

### 4.4. Statistical Analysis

**Figure 5:**
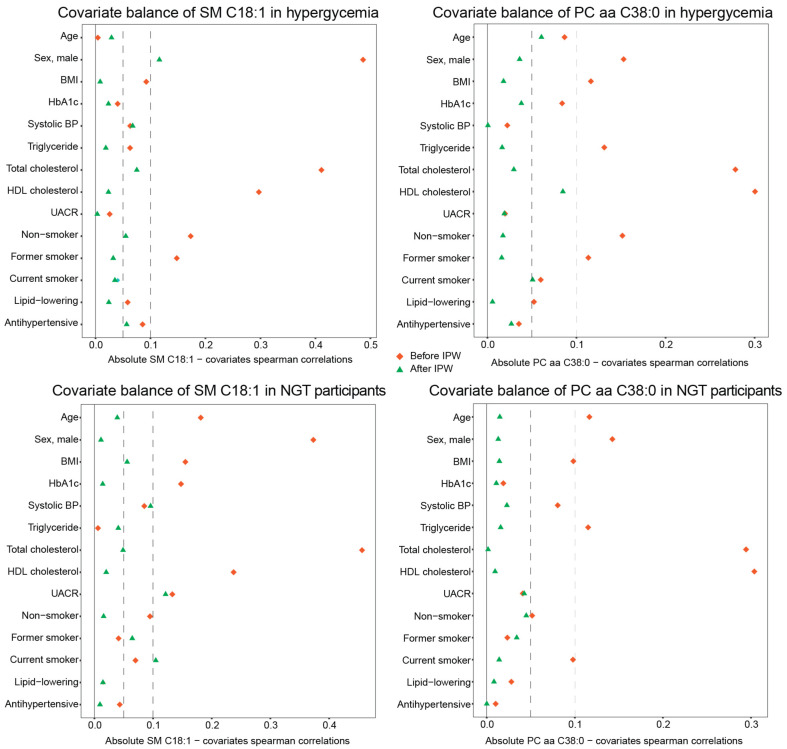
Inverse probability weighting improves metabolite–covariate balance. The absolute values of Spearman’s correlation coefficients for SM C18:1 or PC aa C38:0 with various covariates before and after IPW in hyperglycemic and NGT individuals of KORA FF4 are shown. The interrupted lines represent 0.05 (**left**) and 0.1 (**right**) absolute value of Spearman’s correlation coefficients. Abbreviations: IPW, inverse probability weighting; SM, sphingomyelin; PC aa, phosphatidylcholine diacyl; NGT, normal glucose tolerance. HbA_1c_, glycated hemoglobin; BP, blood pressure; UACR, urinary albumin-to-creatinine ratio.

IPW for continuous exposures of the generalized propensity score approach was applied to reduce the confounding effects and provide a more reliable estimate of metabolite–outcome associations in participants of the KORA FF4 study [56]. The IPW-adjusted analysis improved the balance between two metabolites and covariates, e.g., all of the absolute Spearman’s correlation coefficients between PC aa C38:0 and covariates were below 0.1, both in hyperglycemic and NGT individuals (Figure 5).

We defined two sets of covariates. The basic model included age, sex, BMI, systolic blood pressure, triglyceride, total cholesterol, HDL cholesterol, and HbA_1c_. The full model was additionally adjusted for smoking status, use of lipid-lowering drugs and antihypertensive medication, and UACR. The values of UACR, HbA_1C_ and triglyceride were natural log-transformed before analysis due to their right-skewed distribution.

Generalized propensity scores were estimated with multivariable linear regression in which each metabolite was regressed on covariates from the full model, respectively [57]. The inverse probability weights for each metabolite were then calculated using the corresponding estimated generalized propensity scores [56]. The balance between each metabolite and covariate before and after IPW was estimated by Spearman’s correlation coefficients. Their imbalance was defined using stringent criteria, i.e., with absolute Spearman’s correlation coefficient greater than 0.05.

Metabolite association with eGFR and CKD in hyperglycemic individuals of KORA FF4 was analyzed with weighted multivariable linear and logistic regression with applying corresponding inverse probability weights, respectively. As a sensitivity analysis, metabolite association with CKD was analyzed in NGT individuals of KORA FF4 with weighted multivariable logistic regression after IPW.

Statistical differences in clinical and metabolic parameters between db/db and WT mice were assessed with the Mann–Whitney *U* test. Differences in tissue-specific concentration of creatinine and two candidate metabolite biomarkers between db/db and WT mice were assessed with Student’s *t*-test.

A two-sided *p*-value < 0.05 was considered statistically significant. All statistical analyses were performed using R version 4.0.3.

## 5. Conclusions

This study provides biological insights into our recent discovery of SM C18:1 and PC aa C38:0 as predictive metabolites for incident CKD in hyperglycemic individuals [9]. The cross-sectional analysis showed that the inverse association of both phospholipids with glomerular filtration in hyperglycemic individuals was independent of systolic blood pressure, cholesterol, triglycerides, HbA_1C_, and UACR. Multiorgan analysis in a well-characterized mouse model of early diabetic nephropathy revealed a possible contribution of lungs, liver, adipose tissue, and adrenal glands in their systemic regulation and CKD progression. As a remarkable example of interdisciplinary collaboration, this human and animal study corroborated our initial discovery and provided insights into a relationship with kidney function and the potential implication of other organs. This study contributes to human validation of SM C18:1 and PC aa C38:0 as new biomarkers for early identification of persons with (pre)diabetes with increased risk of CKD and serves as a step ahead towards risk stratification and improved targeted screening programs for CKD. In-depth molecular phenotyping of these novel metabolite predictors of CKD is warranted.

## Figures and Tables

**Table 1:** Characteristics of the KORA FF4 participants according to their hyperglycemic status. Mean ± standard deviation is provided for quantitative variables if not indicated otherwise. *p*-values express the difference between CKD cases and non-CKD controls in hyperglycemic and NGT participants, respectively. *p*-values were calculated by univariate logistic regression if not indicated otherwise. *p*-values shown in bold represent statistical significance at the 0.05 level. Abbreviations: CKD, chronic kidney disease; HbA_1C_, glycated hemoglobin; HDL, high-density lipoprotein; LDL, low-density lipoprotein; NGT, normal glucose tolerance; 2-h glucose, 2-h post-load glucose; BP, blood pressure; eGFR, estimated glomerular filtration rate; UACR, urinary albumin-to-creatinine ratio.

Clinical Variables	Hyperglycemic Participants		NGT Participants			
	CKD *n* = 83	Non-CKD *n* = 427	*p*-Value	CKD *n* = 85	Non-CKD *n* = 1312	*p*-Value
Age, years	74.36 ± 7.66	64.32 ± 10.53	**1.003 × 10^−12^**	72.05 ± 8.23	55.47 ± 10.53	**3.255 × 10^−27^**
Sex, male, %	49.4	57.61	1.686 × 10^−^^1^	48.24	43.9	4.361 × 10^−^^1^
BMI, kg/m^2^	29.25 ± 4.3	30.16 ± 5.02	1.228 × 10^−^^1^	28.11 ± 4.94	26.52 ± 4.37	**1.415 × 10^−3^**
HbA_1C_ (%)	5.74 ± 0.42	5.73 ± 0.54	7.552 × 10^−^^1^	5.56 ± 0.32	5.3 ± 0.32	**7.958 × 10^−12^**
Fasting glucose, mg/dl	112.55 ± 20.44	111.3 ± 16.37 ^b^	6.440 × 10^−^^1^	96.79 ± 7.68	94.02 ± 7.3	**1.130 × 10^−3^**
2-h glucose, mg/dl	164.43 ± 38.98 ^b^	160.63 ± 46.66 ^b^	3.724 × 10^−^^1^	103.16 ± 21.18	95.89 ± 19.9	**3.232 × 10^−3^**
Systolic BP, mmHg	120.31 ± 22.27	124.78 ± 18.03	**4.847 × 10^−2^**	116.65 ± 18.23	115.85 ± 16.06	6.617 × 10^−^^1^
Diastolic BP, mmHg	68.27 ± 11.15	74.93 ± 10.55	**5.054 × 10^−7^**	69.41 ± 10.14	73.06 ± 8.95	**3.048 × 10^−4^**
Triglyceride, mg/dl ^a^	121.11 (93.44–157.4)	128 (92.98–178.27)	9.711 × 10^−^^1^	109 (78–143.79)	93 (70–127.46)	**1.492 × 10^−2^**
Total cholesterol, mg/dl	208.58 ± 41.45	220.93 ± 42.16	**1.533 × 10^−2^**	211.48 ± 43.58	218.59 ± 37.7	9.597 × 10^−^^2^
HDL cholesterol, mg/dl	61.12 ± 18.42	59.78 ± 17.54	5.303 × 10^−^^1^	65.63 ± 18.42	68.57 ± 18.75	1.612 × 10^−^^1^
LDL cholesterol, mg/dl	126.3 ± 35.49	140.65 ± 37.2	**1.456 × 10^−3^**	130.94 ± 37.34	135.83 ± 34.05	2.025 × 10^−^^1^
Creatinine, mg/dl	1.24 ± 0.21	0.89 ± 0.15	**3.916 × 10^−21^**	1.25 ± 0.28	0.86 ± 0.16	**6.345 × 10^−31^**
eGFR, mL/min/1.73 m²	50.5 ± 7.87	81.33 ± 11.9	**3.645 × 10^−47 c^**	50.97 ± 8.01	86.92 ± 12.69	**5.563 × 10^−54 c^**
UACR, mg/g ^a^	9.76 (5.73–26.07)	5.43 (3.39–9.86)	**1.180 × 10^−7^**	7.33 (4.44–15.38)	4.26 (2.94–7.07)	**1.604 × 10^−8^**
Smoking, %			**7.394 × 10^−3^**			**8.080 × 10^−5^**
Nonsmoker	55.42	43.79	Ref.	40	41.54	Ref.
Former smoker	40.96	42.39	2.789 × 10^−^^1^	56.47	40.4	1.086 × 10^−^^1^
Current smoker	3.61	13.82	**1.028 × 10^−2^**	3.53	18.06	**8.628 × 10^−3^**
Medication usage, %						
Lipid-lowering	34.94	22.48	**1.684 × 10^−2^**	32.94	7.7	**2.377 × 10^−12^**
Antihypertensive	84.34	47.07	**1.367 × 10^−8^**	69.41	19.97	**2.272 × 10^−19^**

^a^ Values are presented as median (25th–75th percentile); ^b^ In the hyperglycemic participants, 2-h glucose values were only available in 68 individuals with CKD and 398 individuals without CKD; one non-CKD individual had no fasting glucose values; ^c^ *p*-values calculated with Mann–Whitney *U* test.

**Table 2:** Associations of the two candidate metabolites with eGFR in hyperglycemic individuals. Regression coefficients with 95% *CI* and *p*-values of weighted multivariable linear regression after inverse probability weighting are shown. The basic model was adjusted for age, sex, BMI, systolic blood pressure, triglyceride, total cholesterol, HDL cholesterol, and HbA_1C_. The full model was additionally adjusted for smoking status, use of lipid-lowering drugs, and antihypertensive medication, and urinary albumin-to-creatinine ratio. *p*-values shown in bold represent statistical significance at the 0.05 level. Abbreviations: *CI*, confidence interval; eGFR, estimated glomerular filtration rate; SM, sphingomyelin; PC aa, phosphatidylcholine diacyl.

Models	SM C18:1		PC aa C38:0	
	Effect Estimate (95% *CI*)	*p*-Value	Effect Estimate (95% *CI*)	*p*-Value
Adjusted imbalanced covariates	−1.51 (−2.92 to −0.1) ^a^	**3.624 × 10^−2^**	−1.82 (−3.04 to −0.59) ^b^	**3.757 × 10^−3^**
Basic model	−1.83 (−2.98 to −0.68)	**1.879 × 10^−3^**	−1.91 (−3.11 to −0.72)	**1.784 × 10^−3^**
Full model	−1.76 (−2.9 to −0.62)	**2.499 × 10^−3^**	−1.81 (−2.99 to −0.63)	**2.607 × 10** **^−3^**

^a^ with adjustments for sex, systolic blood pressure, total cholesterol, smoking status, and use of antihypertensive medication; ^b^ with adjustments for age, HDL cholesterol, and smoking status.

**Table 3:** Associations of the two candidate metabolites with CKD in hyperglycemic and NGT individuals. Odds ratios (*OR*s) with 95% *CI* and *p*-values of weighted multivariable logistic regression after inverse probability weighting are shown. The basic model was adjusted for age, sex, BMI, systolic blood pressure, triglyceride, total cholesterol, HDL cholesterol, and HbA_1C_. The full model was additionally adjusted for smoking status, use of lipid-lowering drugs, and antihypertensive medication, and urinary albumin-to-creatinine ratio. *p*-values shown in bold represent statistical significance at the 0.05 level. Abbreviations: *CI*, confidence interval; CKD, chronic kidney disease; SM, sphingomyelin; PC aa, phosphatidylcholine diacyl; NGT, normal glucose tolerance.

Metabolites	Models	NGT Participants		Hyperglycemic Participants	
		*OR* (95% *CI*)	*p*-Value	*OR* (95% *CI*)	*p*-Value
**SM C18:1**	Adjusted imbalance covariates	0.96 (0.77–1.21) ^a^	7.233 × 10^−^^1^	1.46 (1.09–1.97) ^b^	**1.169 × 10^−2^**
	Basic model	1.05 (0.82–1.35)	6.986 × 10^−^^1^	1.93 (1.38–2.78)	**2.251 × 10^−4^**
	Full model	1.14 (0.86–1.51)	3.733 × 10^−^^1^	1.99 (1.37–2.96)	**4.482 × 10** **^−4^**
**PC aa C38:0**	Adjusted imbalance covariates	0.98 (0.78–1.23) ^c^	8.438 × 10^−^^1^	1.61 (1.2–2.17) ^d^	**1.487 × 10^−3^**
	Basic model	1.12 (0.87–1.46)	3.752 × 10^−^^1^	1.68 (1.24–2.29)	**8.723 × 10^−4^**
	Full model	1.19 (0.91–1.58)	2.142 × 10^−^^1^	1.71 (1.23–2.41)	**1.578 × 10^−3^**

^a^ with adjustments for BMI, systolic blood pressure, smoking status, and urinary albumin-to-creatinine ratio; ^b^ with adjustments for sex, systolic blood pressure, total cholesterol, smoking status, and use of antihypertensive medication; ^c^ no additional adjustment; ^d^ with adjustments for age, HDL cholesterol, and smoking status.

**Table 4:** Phenotypic and metabolic variables in db/db and wild type mice after 5 weeks of a high-fat diet. Values are mean ± SD. *p*-values were calculated by Mann–Whitney *U* test. *p*-values shown in bold represent statistical significance at the 0.05 level. Abbreviations: db/db, leptin receptor-deficient mouse model; HDL, high-density lipoprotein; LDL, low-density lipoprotein.

Clinical Variables	db/db Mice *n* = 10	Wild Type Mice *n* = 10	*p*-Value
Body weight, g	47.87 ± 2.37	21.97 ± 0.58	**1.796 × 10^−4^**
Kidney weight, g	0.20 ± 0.02	0.16 ± 0.02	**2.057 × 10^−4^**
Liver weight, g	2.56 ± 0.3	1.02 ± 0.09	**1.083 × 10^−5^**
Heart weight, g	0.14 ± 0.01	0.12 ± 0.01	**4.871 × 10^−4^**
Blood glucose, mg/dL	421.60 ± 41.24	106.7 ± 16.88	**1.806 × 10^−4^**
Plasma insulin, µg/L	7.76 ± 2.33	1.03 ± 0.4	**1.083 × 10^−5^**
Triglyceride, mg/dL	224.78 ± 106.51	122.24 ± 24.52	5.869 × 10^−^^2^
Total cholesterol, mg/dL	153.24 ± 16.14	100.58 ± 12.16	**1.817 × 10^−4^**
HDL cholesterol, mg/dL	125.28 ± 13.12	84.28 ± 8.65	**1.083 × 10^−5^**
LDL cholesterol, mg/dL	18.76 ± 3.67	14.5 ± 2.08	**8.127 × 10^−3^**
C-reactive protein, mg/L	13.12 ± 3.27	5.36 ± 1.12	**1.786 × 10^−4^**
Plasma creatinine ^a^, mg/dL	0.05 ± 0.01	0.08 ± 0.01	**2.076 × 10^−4^**
Plasma albumin, g/dL	3.10 ± 0.34	2.56 ± 0.13	**5.509 × 10^−4^**

^a^ The clinical chemistry-measured creatinine values are reported here.

**Table 5:** Biofluid- and tissue-specific trends of creatinine and two candidate CKD metabolites. Results of *t* statistic and *p*-values of two biofluids and six tissues between 10 db/db and 10 WT mice on a high-fat diet are shown. *p*-values shown in bold represent statistical significance at the 0.05 level. Abbreviations: CKD, chronic kidney disease; db/db, leptin receptor-deficient mouse model; WT, wild type mice; SM, sphingomyelin; PC aa, phosphatidylcholine diacyl.

Tissue	Creatinine		SM C18:1		PC aa C38:0	
	*t* Statistic	*p*-Value	*t* Statistic	*p*-Value	*t* Statistic	*p*-Value
Plasma	−5.68	**2.284 × 10^−5^**	4.71	**3.160 × 10^−4^**	0.35	7.327 × 10^−^^1^
Urine ^a^	−9.20	**9.396 × 10^−8^**	−2.39	**4.193 × 10^−2^**	−4.56	**4.516 × 10^−4^**
Liver	−9.21	**5.298 × 10^−8^**	6.00	**1.288 × 10^−5^**	0.19	8.499 × 10^−^^1^
Lung	−3.54	**2.531 × 10^−3^**	2.46	**2.440 × 10^−2^**	3.60	**2.173 × 10^−3^**
Adrenal glands ^b^	1.33	2.098 × 10^−^^1^	0.16	8.745 × 10^−^^1^	4.11	**9.695 × 10** **^−4^**
Adipose tissue ^c^	−0.49	6.308 × 10^−^^1^	−3.70	**1.763 × 10 ^−3^**	−2.36	**3.856 × 10** **^−2^**
Cerebellum	−0.37	7.164 × 10^−^^1^	1.18	2.543 ×10^−^^1^	1.46	1.605 × 10^−^^1^
Testis	2.05	5.560 × 10^−^^2^	−0.52	6.069 × 10^−^^1^	−0.28	7.849 × 10^−^^1^

^a^ For SM C18:1, *n* = 7 in db/db, *n* = 9 in WT. For PC aa C38:0 and creatinine, *n* = 9 in db/db, *n* = 9 in WT. ^b^ For creatinine, *n* = 9 in db/db, *n* = 9 in WT. ^c^ For creatinine, *n* = 9 in db/db, *n* = 10 in WT.

## Data Availability

The KORA FF4 data sets are not publicly available because of data protection agreements but can be provided upon request through the KORA-PASST (Project application self-service tool, www.helmholtz-muenchen.de/kora-gen).
